# Proteomic Analysis of Duodenal Tissue from *Escherichia coli* F18-Resistant and -Susceptible Weaned Piglets

**DOI:** 10.1371/journal.pone.0127164

**Published:** 2015-06-08

**Authors:** Zhengchang Wu, Riwei Xia, Xuemei Yin, Yongjiu Huo, Guoqiang Zhu, Shenglong Wu, Wenbin Bao

**Affiliations:** 1 Key Laboratory for Animal Genetics, Breeding, Reproduction and Molecular Design of Jiangsu Province, College of Animal Science and Technology, Yangzhou University, Yangzhou, 225009, P. R. China; 2 College of Veterinary Medicine, Yangzhou University, Yangzhou, Jiangsu, P. R. China; China Agricultrual University, CHINA

## Abstract

Diarrhea and edema disease in weaned piglets due to infection by *Escherichia coli* F18 is a leading cause of economic loss in the pig industry. Resistance to *E*. *coli* F18 depends on expression of receptors on intestinal epithelial cells, and individual immunity. This study was conducted in Sutai pig *E*. *coli* F18-resistant and -susceptible full sib-pair individuals, identified on the basis of resource populations and verification of adhesion assays. The molecular mechanism underlying *E*. *coli* F18 resistance was investigated through analysis of the expression of *E*. *coli* F18 receptor associated and innate immunity proteins, using proteomics and bioinformatics techniques. Two-dimensional electrophoresis analysis revealed a total of 20 differentially expressed proteins in *E*. *coli* F18-resistant and -susceptible groups (10 upregulated and 10 downregulated). A total of 16 differentially expressed proteins were identified by MALDI TOF/TOF mass spectral analysis. According to gene ontology and pathway analysis, differentially expressed proteins were mainly involved in cell adhesion, immune response and other biologically relevant functions. Network analysis of interactions between differentially expressed proteins indicated a likelihood of their involvement in *E*. *coli* F18 infection. The expression levels of several important proteins including actin beta (ACTB), vinculin (VCL), heat stress proteins (HSPs) and transferrin (TF) in *E*. *coli* F18-resistant and -susceptible individuals were verified by Western blotting, supporting the identification of ACTB, VCL, HSPs and TF as promising candidate proteins for association with *E*. *coli* F18 susceptibility.

## Introduction

*Escherichia coli* strain F18 is the main pathogenic bacterium responsible for post-weaning diarrhea (PWD) and edema disease (ED) in piglets. The pathogenicity of *E*. *coli* F18 depends on expression of receptors for the *E*. *coli* F18 pilus on the brush border of piglet alvine epithelial cells. Piglets lacking this receptor exhibit resistance to *E*. *coli* F18 [1]. However, specific *E*. *coli* F18 binding receptors in intestinal tracts of piglets have not yet been identified.

Many studies have focused on the molecular mechanism controlling the adhesion of *E*. *coli* F18 to its receptor. Using the candidate gene approach and linkage analysis, Vogeli et al. suggested that the alpha (1,2)-fucosyltransferase (*FUT1*) gene on chromosome 6qll is a candidate gene for controlling adhesion to the *E*. *coli* F18 receptor. Furthermore, it was observed that G to A mutation of the M307 site in the *FUT1* gene was related to receptor formation and could be used as a genetic marker for screening. Specifically, the AA genotype was associated with *E*. *coli* F18 resistance, while the AG and GG genotypes were associated with susceptibility [2]. However, analysis of *FUT1* gene polymorphism in dozens of local pig breeds in China revealed that the AA genotype associated with *E*. *coli* F18 resistance is not represented [3], which suggested that it is not feasible to select for the *FUT1* resistance genotype (AA genotype) directly from Chinese native pig breeds. It is well known that Chinese local pig breeds generally exhibit characteristic strong resistance and tolerance to disease, suggesting that indigenous Chinese pigs and foreign pig breeds have different molecular mechanisms and physiological functions related to the formation and structure of receptor molecules or innate and adaptive immunity against *E*. *coli* F18 infection. Therefore, it is necessary to seek effective molecular markers for *E*. *coli* F18 resistance in Chinese pig breeds.

A comprehensive understanding of the regulation and range of gene expression is very important to elucidate the biological role of the encoded protein. Changes in gene expression configuration can also provide evidence concerning regulatory mechanisms, cellular functions and biochemical pathways [4]. Accordingly, researcher interest has focused on the identification and characterization of the functional protein products encoded by the genome, whether independent or organized as a tissue [5]. Protein-based methods are important because they characterize translational regulation and post-translational modifications. Among these methods, two-dimensional electrophoresis (2DE) and matrix-assisted laser desorption/ionization-time of flight mass spectrometry (MALDI-TOF MS) monitoring of proteome levels are considered to be very useful tools.

In order to elucidate the genetic basis of *E*. *coli* F18-resistance in Chinese local pig breeds, Sutai pigs were selected as the experimental animal. Sutai pig is the hybridization product of Duroc and Taihu pigs after 15 years of cross-breeding. In 1999, it was approved by National Committee of Livestock and Poultry Species as a new breed. Using a small number individuals of AG genotype (9.2%) in *FUT1* gene detected from Sutai pigs, our group conducted proper selection and assortative mating. After five years of continuous molecular selection and breeding, two resource populations of Sutai pigs were established, one being *E*. *coli* F18-resistant (AA type) and the other *E*. *coli* F18-sensitive (AG or GG type). *Escherichia coli* are a group of Gram-negative flagellated bacteria that normally reside and multiply in the intestinal tract of animals. Veterinary pathology experiments have demonstrated that the duodenum and jejunum are main sites of *E*. *coli* F18 strain colonization and replication. In previous studies, duodena were collected for *E*. *coli* F18 adhesion and high-throughput sequencing studies [6,7]. The present study used proteomics and bioinformatics techniques to compare differential protein expression in duodenal tissues of weaned Sutai piglets in a complete sib-pair group showing sensitivity and resistance to *Escherichia coli* F18, respectively. Identification and analysis of all differentially expressed proteins was performed by mass spectrometry (MALDI/TOF/TOF-MS/MS), to elucidate the differences in molecular mechanisms of intestinal epithelial cell receptor expression and intestinal innate immunity in *E*. *coli* F18-resistant and susceptible individuals. The differential expression of proteins in *E*. *coli* F18-resistant and -susceptible individuals was further validated by Western blot analysis. The purpose of the study was to explore the receptor proteins for *E*. *coli* F18 and the genetic basis of *E*. *coli* F18-resistance in Sutai weaned piglets. Moreover, since Sutai is a synthetic breed with 50% Chinese Taihu origin, this study not only identified several candidate protein by proteomics analysis, but also these proteins could be further validated in Chinese local pig breeds in order to improve *E*. *coli* F18 resistance breeding in such Chinese local pig breeds.

## Materials and Methods

### Ethics statement

The animal study proposal was approved by the Institutional Animal Care and Use Committee (IACUC) of the Yangzhou University Animal Experiments Ethics Committee with permit number SYXK(Su) IACUC 2012–0029. All piglet experimental procedures were performed in accordance with the Regulations for the Administration of Affairs Concerning Experimental Animals approved by the State Council of the People’s Republic of China.

### Experimental materials

**Fig 1:**
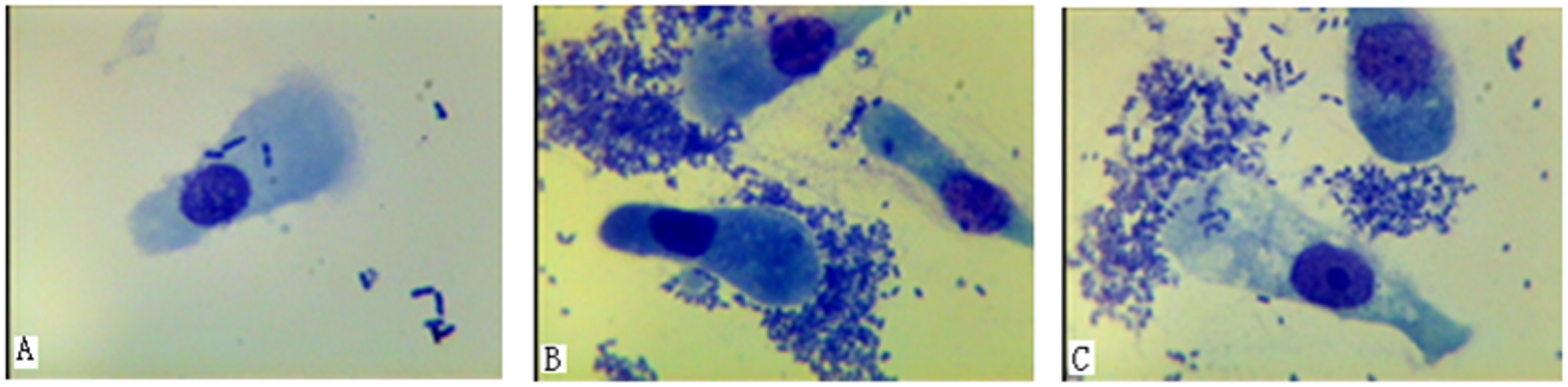
Adhesion test of intestinal epithelial cells from *E*. *coli* F18-resistant and -sensitive piglets. (A) represents *E*. *coli* F18-resistant piglets; (B) and (C) represent *E*. *coli* F18-sensitive piglets. Images were recorded using an oil immersion lens at 1000 × magnification.

Forty full-sib healthy individuals from eight families were selected from the *E*. *coli* F18 disease-resistant basic breeding colony of Sutai pigs in the Suzhou Sutai Pig Breeding Center, bred following complete screening of *E*. *coli* F18-susceptible and -resistant individuals. Further analysis and verification of *E*. *coli* F18 resistance/susceptibility was conducted using the adhesion test for intestinal epithelial cells: the F18ab fimbriae standard strain 107/86 (O139: K12: H1) was provided as a gift by the veterinary laboratory at the Institute of Microbiology, University of Pennsylvania. Expression of recombinant *E*. *coli* F18 fimbriae was from the vector pET22b carrying the F18 operon. Expression of F18ac fimbriae that contained the *fed* operon of recombinant *E*. *coli* r*E*.*coli*1534 was optionally induced by IPTG, and the surface expression of the F18ab fimbriae FedF subunit of *E*. *coli* pnirBMisL-fedF was constructed and stored [8,9]. Anaerobic bacteria were washed with phosphate buffer saline (PBS), and the bacterial concentration was adjusted to approximately 1 × 10^9^ CFU/mL. After adding 1% mannose (w/v) to 0.5 mL of wild-type or recombinant bacteria, the suspensions were incubated at 37°C for 30 min, mixed with 0.5 mL of small intestinal cells, incubated at 37°C for 30 min, and finally centrifuged at 1,000 rpm (201 × *g*) for 5 min. At this time, 50 μL of the preparation was extracted after resuspension in PBR (0.24 g/L KH_2_PO_4_, 1.44 g/L Na_2_HPO_4_, 0.42 g/L KCl, 9 g/L NaCl, 0.25 g/L CaCl_2_). The mixture was deposited on a glass slide, air dried, heat-fixed, and stained with methylene blue for 3–5 min. Then, bacterial association with the intestinal cells was visualized microscopically [10]. Fifteen centimeters of duodenal tissue were obtained according to the approach that Alwan et al. used for the isolation and preparation of intestinal epithelial cells [11]. The adhesion of bacteria was evaluated quantitatively by counting the mean number of bacteria adhering along a 50 μm villous brush border at 20 randomly selected sites for each piglet. Adhesion of less than 5 and more than 30 bacteria per 250 mm brush border length was noted as resistant or susceptible, respectively [12]. In all piglets analyzed by the binding assay, we strictly identified piglets displaying no adherence with F18ab-expressing fimbriae of the standard *ETEC* strain as *E*. *coli* F18-resistant individuals (Fig 1A), in contrast, piglets displaying a large number of adherence as *E*. *coli* F18- susceptible individuals (Fig 1B and 1C). By this process, we finally obtained four pairs of *E*. *coli* F18-susceptible and *E*. *coli* F18-resistant individuals for proteomics analysis. The animals were allowed access to food and water ad libitum under normal conditions. Around the time of weaning, when the piglets were 28 days old, most vulnerable to ETEC F18 infection, they were humanely sacrificed by an intravenous injection of sodium pentobarbital as necessary to ameliorate suffering. In previous studies, duodenal tissue was selected for *E*.*coli* F18 adhesion and high-throughput sequencing [6,7]. In view of the data available from these studies, we chose to also use the duodenal tissue in this study. About 100 mg of duodenal tissue was removed and the scraped epithelium of the duodenum was placed into 1.5 mL nuclease-free Eppendorf tubes, frozen in liquid nitrogen and stored at -80°C until further use.

### Protein extraction and quantification

Samples were washed with normal saline, homogenized in liquid nitrogen and 100 μL lysis buffer (9.5 M urea, 4% CHAPS, 65 mM DTT and ampholyte with 2% carrier (pH 3-10NL, GE healthcare, Piscataway, NJ, USA)), and an enzyme inhibitor cocktail (Roche, Basel, Switzerland) was added. A small sample of the homogenized tissue was subjected to ultrasonication (eight periods of 6 s at 80 W at 10 s intervals). The homogenate was incubated on ice for 1 h and centrifuged for 20 min at 21,480 × *g* (Eppendorf Centrifuge 5810R, Baltimore, MD, USA). The supernatant was collected for analysis. “Bio-Rad Protein assay” reagent (Bio-Rad Laboratories, CA, USA) was used for protein quantification.

### Two-dimensional gel electrophoresis and data analysis

Experimental 2-DE was performed according to the IPGphor method [13]. Protein samples (100 μL) were subjected to isoelectric focusing (IEF) using hydrated gel strips (13 cm, pH 3–10) (Electrophoresis conditions were 30 V for 12 h, 500 V for 1 h, 1000 V for 1 h, 8000 V for 6 h and 500 V for 4 h). IPG gel strips were immediately placed into 2 mL balance buffer A (6 M urea, 0.05 M Tris-HCl (pH 8.8), 2% SDS, 30% (w/v) glycerol and trace bromophenol blue were mixed and stored at -20°C. 1% DTT was added before use) and 2 mL balance buffer B (6 M urea, 0.05M Tris-HCl (pH 8.8), 2% SDS and 30% (w/v) glycerol) were added. Samples were stored at -20°C. Samples were equilibrated for 15 min and 1.25% iodoacetamide was added before use. Equilibrated IPG gel strips were used for the second-dimension SDS-PAGE electrophoresis for 30 min at 15 mA/gel. The current was then increased to 30 mA/gel. SDS polyacrylamide gels were stained with silver nitrate [14] and images were acquired and analyzed using a GS-710 optical density scanner with Imagemaster software (GE Healthcare, Piscataway, NJ, USA). Three replicate 2-DE spectra were analyzed to identify statistically significant differences in protein expression (quantitative ratio between two groups of samples > 1.5).

### Enzymolysis and mass spectrum analysis of differentially expressed proteins

Spots of differentially expressed proteins were cut from the gels and decolorized using 30 mM K_3_Fe(CN)_6_ and 100 mM Na_2_S_2_O_3_ (1:1 v/v), freeze-dried and incubated for approximately 20 h at 37°C in sequencing-grade trypsin solution (Promega, Madison, WI, USA). The enzymolysis liquid was removed into a separate tube and 100 μL 60% ACN/0.1% TFA were added into the original tubes which were subjected to ultrasonic processing for 15 min. The resultant solutions were combined and freeze-dried. Samples were desalted using Ziptips (Millipore, Bedford, MA, USA) according to the manufacturer’s instructions.

Samples were mixed with 5 mg/mL HCCA matrix at a ratio of 1:1 for second-stage mass spectral analysis (MS/MS) using a 4800 Plus MALDI TOF/TOF Analyzer (Applied Biosystems, USA) with the following conditions: acceleration voltage 2 kV; PMF mass scanning range 800–4,000 Da; parent ions with signal-noise ratio > 50. Peptide mass fingerprint spectra were analyzed with Data Explore Software to generate corresponding protein peptide sequences. For protein identification, the MS/MS spectra were searched using Mascot software (Matrix Science, London, United Kingdom; http://www.matrixscience.com) using the genome data of *Sus scrofa* from NCBInr (http://www.ncbi.nih.gov).

### Bioinformatics analysis of differentially expressed proteins

Differentially expressed proteins corresponding to porcine genes were defined as differential genes. Gene Ontology (GO) Analysis (http://www.geneontology.org/) and Pathway Analysis (http://www.genome.jp/kegg/) of differential genes were carried out to identify all gene-related functions. Using R software (http://www.r-project.org/), Fisher’s exact testing and multiple hypothesis testing were carried out to determine the statistical significance (*P*-value) and false discovery rate (FDR) of each differential gene-involved GO Pathway, based on the method of Benjamini and Hochberg [15]. Differential expressed genes within a significant GO Pathway were screened; significant screening standards were defined as *P* < 0.05 and FDR < 0.05. The interaction between differential genes and gene products was identified using the KEGG database.

### Real-time PCR analysis

Total RNA (500 ng) was reverse transcribed in a final reaction volume of 10 μL using a PrimerScript RT reagent kit (TaKaRa Biotechnology Dalian Co., Ltd). The reactions contained 5 μL 5 × PrimerScript Buffer, 0.5 μL PrimerScript RT Enzyme Mix I, 0.5 μL Oligo (dT), 0.5 μL random hexamers and RNase-free H_2_O. The cDNA was synthesized at 37°C for 15 min followed by a termination step at 85°C for 5 s and then stored at −20°C. Real-time PCR amplification was performed in 20 μL reaction mixtures containing 1 μL cDNA, 0.4 μL 50 × ROX Reference Dye II, 10 μL 2 × SYBR Green Real-time PCR Master Mix, 7.8 μL dd H_2_O, 0.4 μL (10 μM) of each pair of specific primers or *GAPDH* primers. All primer sequences are shown in S1 Table. Real-time PCR was performed on an ABI 7500 system (Applied Biosystems, Foster City, CA, USA). PCR cycling parameters were: 95°C for 15 s; and then 95°C for 5 s followed by 62°C for 30 s for 40 cycles. Dissociation curve analysis was performed at the end of the 40 cycles to verify PCR product identity. Each sample was tested three times to obtain average data.

### Western blot analysis

To confirm the differential expression of proteins identified by 2-DE in *E*. *coli* F18-resistent and -susceptible individuals, total proteins were extracted using a NE-PER kit (Nuclear and Cytoplasmic Extraction Reagents, Thermo Fisher Scientific Inc.) according to the manufacturer’s protocol. Protein levels were normalized using a BCA kit (Thermo Fisher Scientific Inc.). SDS-PAGE conditions were, 10 μL protein loaded to a 10% gel run at 120 V for 90 min. For western blotting, proteins were transferred to PVDF membranes and immunoblotted with primary antibodies against ACTB (0.19 mg/mL, 42 KDa), TF (0.26 mg/mL, 78.9 KDa), HSP27 (0.47 mg/mL, 22.9 KDa) or VCL (0.23 mg/mL, 116.9 KDa) (Abmart, Inc.). The secondary antibody was HRP conjugated goat anti-rabbit IgG (Beijing ComWin Biotech Co., Ltd., 1:2500).

## Results

### Electrophoretic analysis of duodenal proteins in *E*. *coli* F18-resistent and -susceptible individuals

Duodenal proteins from *E*. *coli* F18-resistent and -susceptible individuals were analyzed by 2-DE. Gel images were obtained with improved reproducibility and higher resolution following silver nitrate staining (S1 Fig). The apparent molecular weights of the majority of protein spots ranged from 14 kDa to 97 kDa, with isoelectric points distributed between 4 and 10. Very few proteins were identified in extremely acidic or alkaline regions.

### Differential display of duodenal proteins in *E*. *coli* F18 -resistant and -susceptible individuals

**Fig 2:**
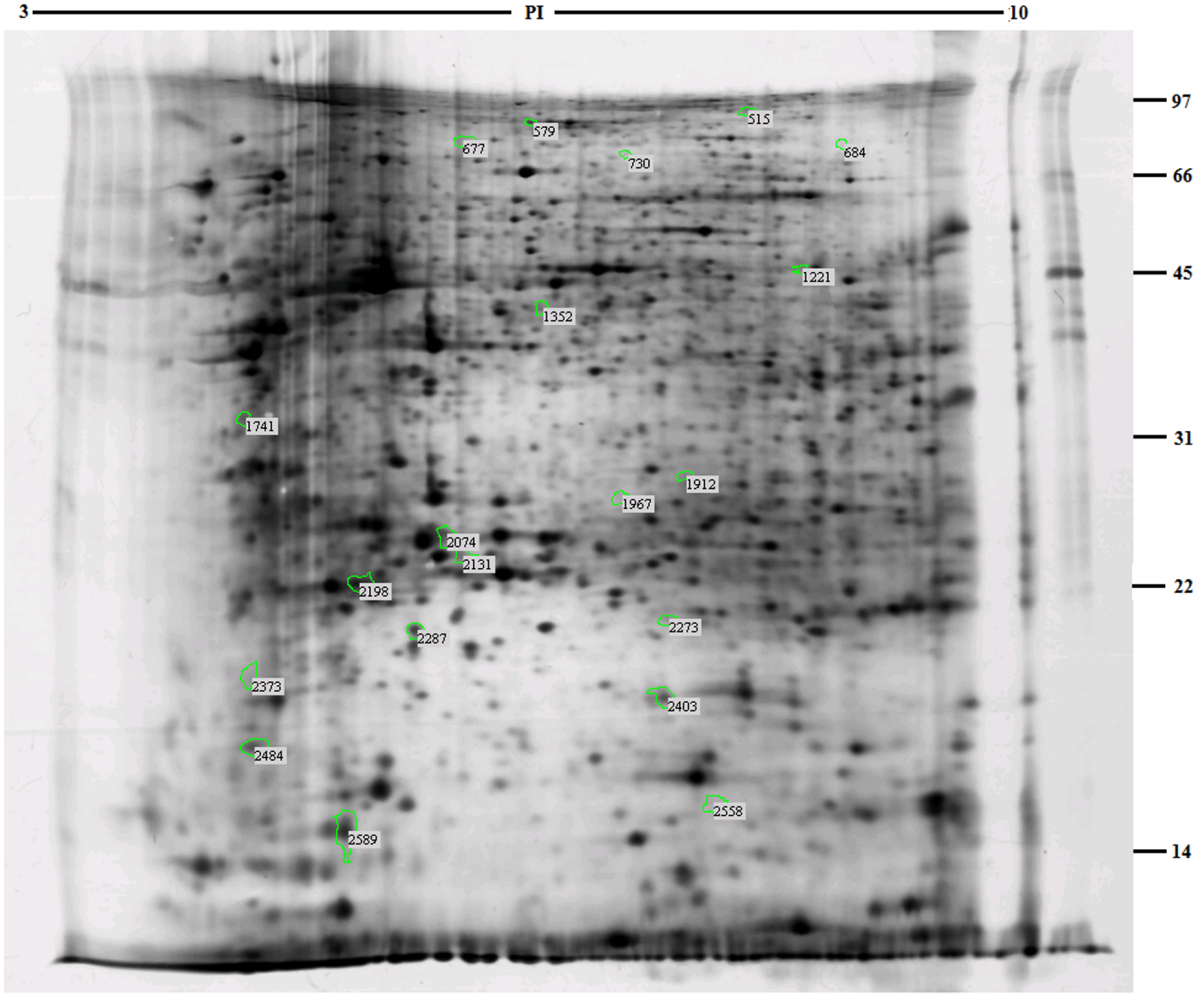
Analysis of two-dimensional electrophoresis highlighting differentially expressed proteins in duodenal tissues of *E*. *coli* F18-resistant and -susceptible individuals.

A total of 20 differential protein spots showed a significant change in expression on analysis with Imagemaster software (Fig 2). The group IDs of these protein spots were 515, 579, 677, 684, 730, 1221, 1352, 1741, 1912, 1967, 2074, 2131, 2198, 2273, 2287, 2373, 2403, 2484, 2558 and 2589, respectively. Among these, 10 spots were highly expressed in the *E*. *coli* F18-resistant group and 10 different spots were highly expressed in the *E*. *coli* F18-susceptible group (S2 Table).

### Mass spectral analysis of differentially expressed duodenal proteins in *E*. *coli* F18-resistant and -susceptible individuals

MALDI-MS/MS analysis was undertaken for the 20 differentially expressed proteins identified by comparison of the two groups. Peptide fingerprint spectra were successfully obtained for 16 protein spots. Target proteins were identified by searching for homologous proteins and peptides in the NCBInr database (http://www.ncbi.nih.gov).

A total of 12 proteins were identified among the 16 differentially expressed protein spots (Table 1). Among these, two groups of spots (2287, 2589, 2484 and 2074, 1967, 1912) represented actin alpha 2 (ACTA2) and albumin (ALB) respectively. Other differentially expressed proteins included four different upregulated proteins in the *E*. *coli* F18-resistant group: transferrin (TF); similar to collapsin response mediator protein-2A (LOC100151886); ribosomal protein SA (RPSA); and similar to AGAP005293-PA (LOC100153507). There were also six upregulated proteins in the *E*. *coli* F18-susceptible group: vinculin (VCL); aconitase 2 (ACO2); actin, alpha cardiac muscle 1 (ACTC1); actin beta (ACTB); heat shock protein 27 kDa (HSP27); and smooth muscle protein 22-alpha (SM22A).

**Table 1:** Differential proteins in duodenal tissues of individuals between the *E*. *coli* F18-resistant group and the susceptible group.

Spot	Protein gi	Protein MW	Protein PI	Protein score	Protein Score C.I%	RNA_nucleotide accession	Gene ID	Symbol	Description	Regulation
2373	194037939	56732.2	4.69	116	100	XM_001924781.1	100153507	LOC100153507	Similar to AGAP005293-PA	2.373
1741	122069666	33021.5	4.8	191	100	----	641351	RPSA	Ribosomal protein SA/37-kDa laminin Receptor precursor/ (LRP)/67-kDa laminin receptor (LR)	2.066
730	194041527	62681.8	5.95	113	100	XM_001927796.1	100151886	LOC100151886	Similar to collapsin Response mediator protein-2A	1.881
579	833800	78954.1	6.73	83	99.988	X12386.1	396996	TF	Transferrin	1.776
2484	254771936	42381	5.23	79	99.97	FJ547477.1	42381	ACTA2	Actin, alpha 2, smooth muscle, aorta	1.646
1967	833798	71361.6	5.92	417	100	X12422.1	396960	ALB	Albumin	1.603
2287	257470979	42381	5.23	183	100	NM_001164650.1	733615	ACTA2	Actin, alpha 2, smooth muscle, aorta	-2.856
677	17979613	117373.7	5.89	139	100	AF165172.1	396974	VCL	Vinculin	-2.395
515	113159	86448.5	8.24	77	99.955	----	396999	ACO2	Aconitase 2, mitochondrial	-2.334
1912	833798	71361.6	5.92	252	100	X12422.1	396960	ALB	Albumin	-2.109
2131	50916342	14268.2	5.94	146	100	AY574049.1	493184	HSP 27	Heat shock protein 27kDa	-1.592
2589	254771936	42381	5.23	300	100	FJ547477.1	42381	ACTA2	Actin, alpha 2, smooth muscle, aorta	-1.589
2403	2984713	10156.2	4.93	152	100	AF053629.1	397021	SM22A	Smooth muscle protein 22-alpha	-1.584
1352	187692583	27588.8	4.98	132	100	EU655628.1	100158242	ACTC1	Actin, alpha, cardiac muscle 1	-1.570
2198	45269029	45162.4	5.55	363	100	AY550069.1	414396	ACTB	Actin, beta	-1.559
2074	833798	71361.6	5.92	358	100	X12422.1	396960	ALB	Albumin	-1.558

“Spot” represents the serial number of a protein in two-dimensional electrophoresis; “Protein MW” represents protein molecular weight; “Protein PI” represents protein isoelectric point; “Protein Score C.I%>95” represents successful identification; The positive value of “Regulation” represents up-regulated proteins in the *E*. *coli* F18-resistant group compared with the susceptible group, the negative value represents down-regulated proteins.

### Gene ontology and pathway analyses of differentially expressed proteins

**Fig 3:**
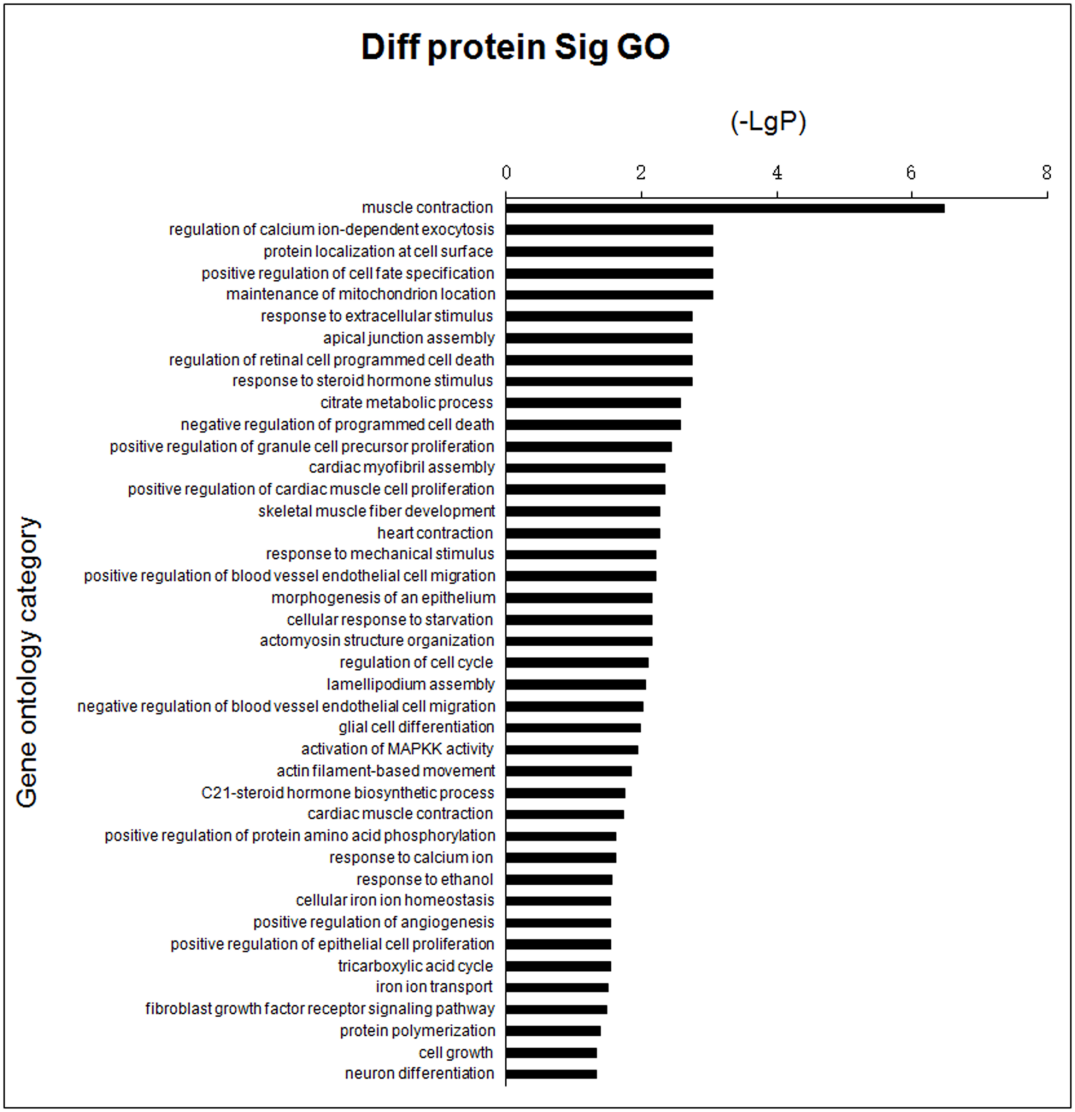
Bar chart showing GO analysis of differentially expressed proteins in groups of *E*. *coli* F18-resistant and -susceptible individuals. Y-axis represents the name of GO; X-axis represents the minus logarithm of p-value (-LgP).

**Fig 4:**
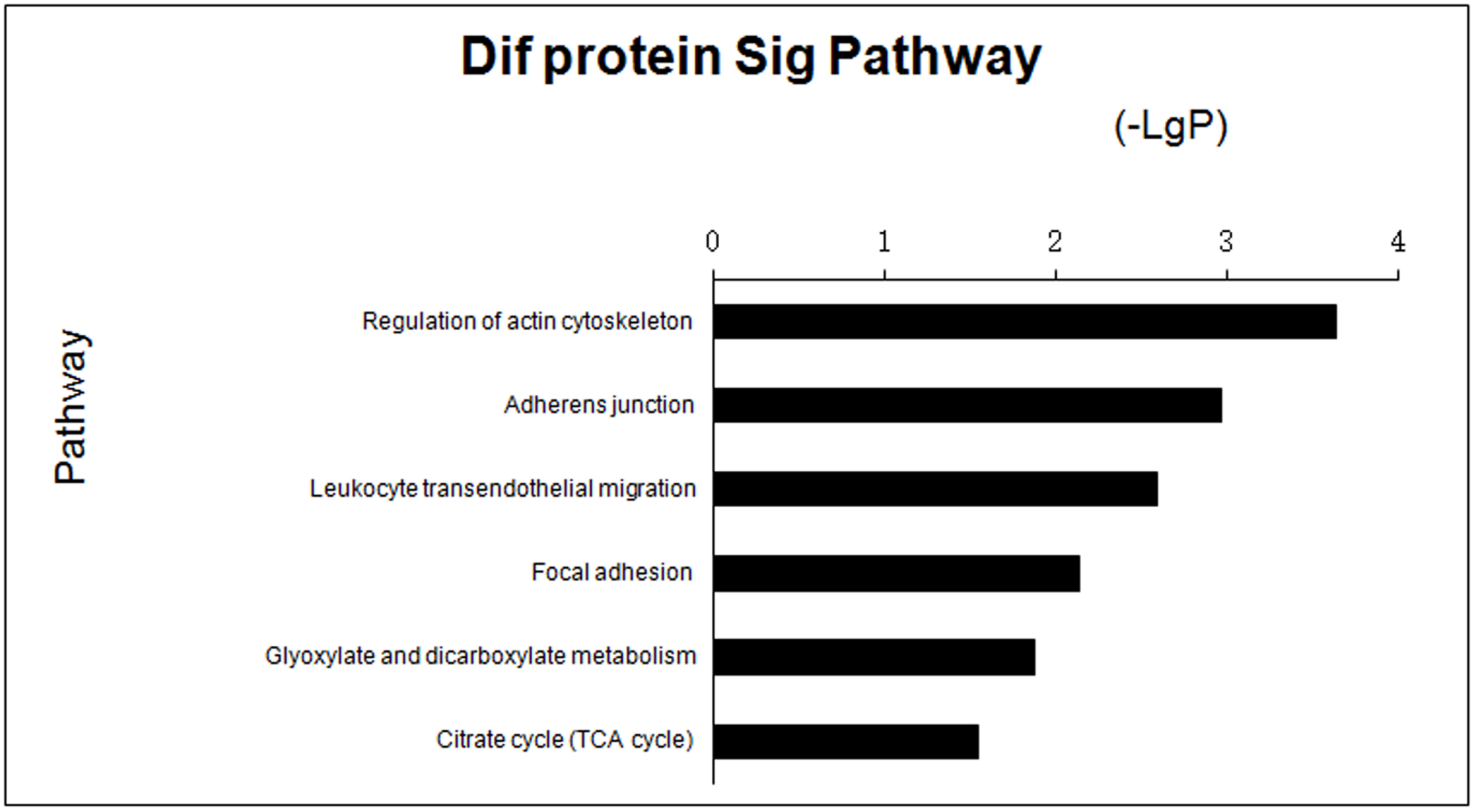
Bar chart showing pathway analysis of differentially expressed proteins in groups of *E*. *coli* F18-resistant and -susceptible individuals. Y-axis represents the name of pathway; X-axis represents the minus logarithm of p-value (-LgP).

GO analysis was used to identify genes with known functions, including muscle contraction, cell surface protein localization, response to extracellular stimuli and activation of MAPKK (Fig 3, S3 Table). Pathways of differential genes corresponding to the differentially expressed proteins identified in the KEGG database included regulation of the actin cytoskeleton, adherens junction formation, leukocyte transendothelial migration and focal adhesion (Fig 4, S4 Table). In general, each GO or pathway included several of the differentially expressed genes, although no differentially expressed gene was limited to one GO or pathway.

### Network diagram analysis of interactions between differentially expressed proteins

**Fig 5:**
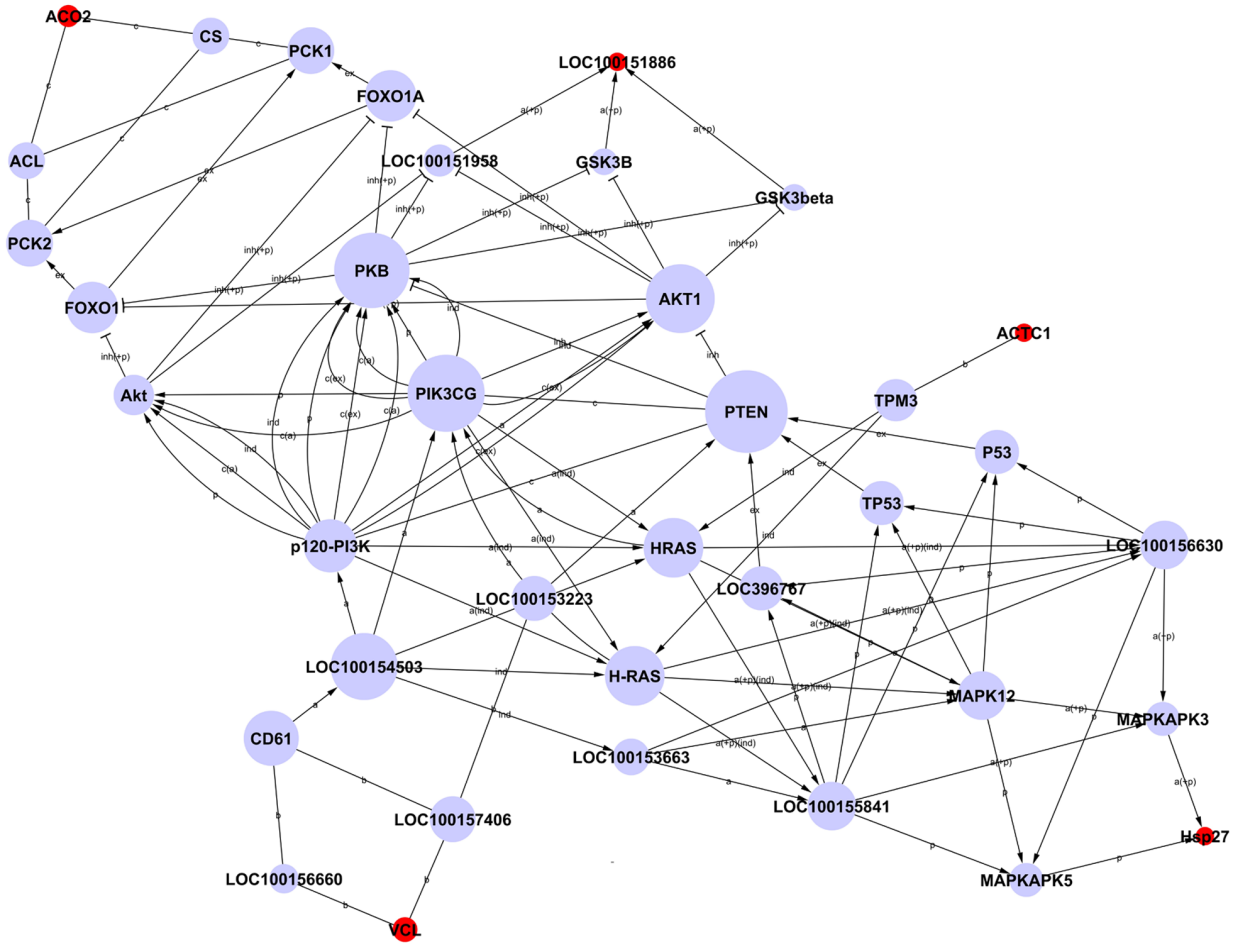
Network of interactions of differentially expressed proteins. Note: Each circle represents a protein (red, differential protein; blue, linking protein). The area of each circle represents the value of degree, i.e. the extent of interactions between one protein and other proteins.

Network diagrams of interactions between differentially expressed proteins were constructed based on the KEGG database information by combining proteins in all significant pathways in order to identify any correlation of target proteins (Fig 5). Due to the small number of differentially expressed proteins identified in this study, and limitations on the information in the database, the network diagram included only five differential proteins, ACO2, LOC100151886, ACTC1, VCL and HSP27 (indicated by red spots in Fig 5), plus connecting proteins. These five proteins were located upstream or downstream of the network. According to the analysis method of graph theory, the nodes most upstream or downstream of a network are of particular importance, which implies that the five differentially expressed proteins considered in the analysis are likely to be related to *E*. *coli* F18 infection to some extent (S5 Table).

### Verification of differentially expressed proteins related to *E*. *coli* F18 infection

**Fig 6:**
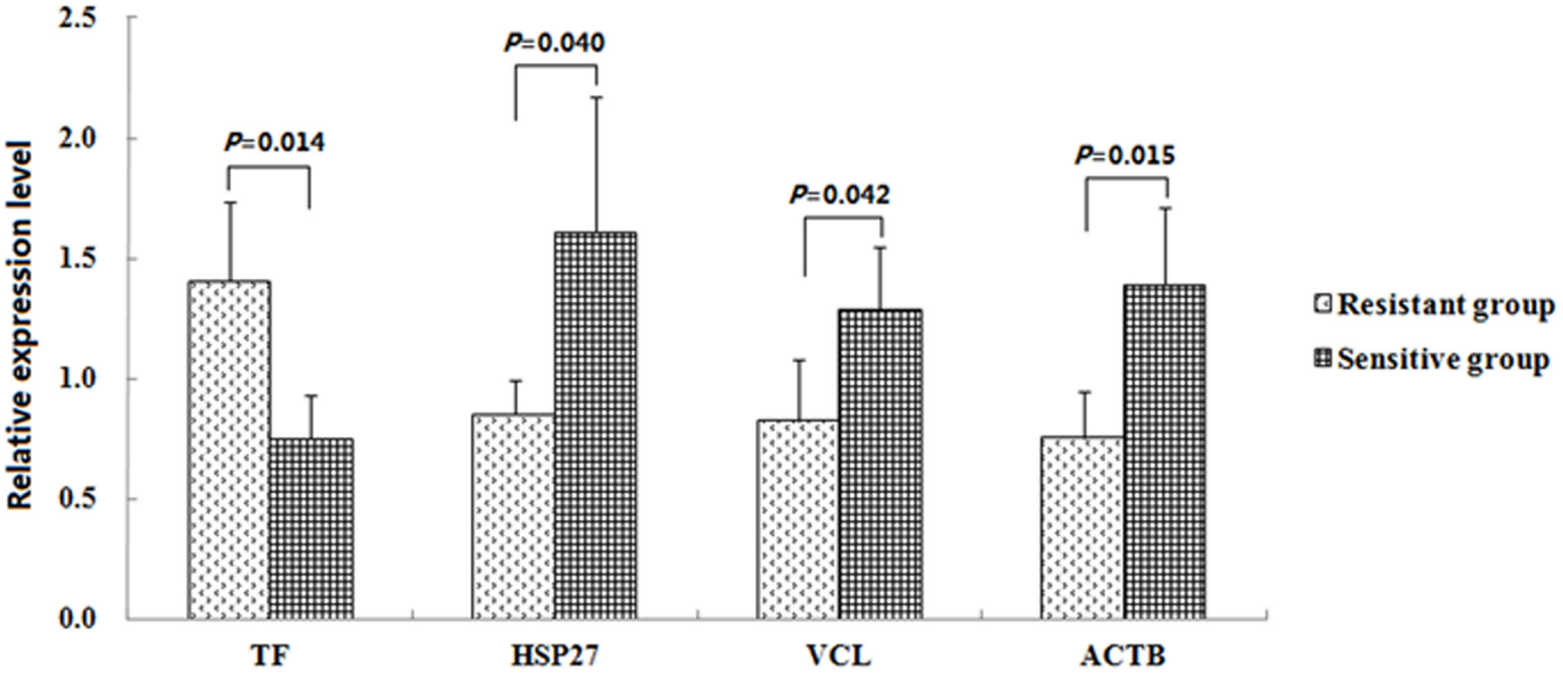
Differential expression of *VCL*, *TF*, *HSP27* and *ACTB* genes in duodenal tissue between *E*. *coli* F18-resistant and -susceptible groups, analyzed by Real-time PCR.

**Fig 7:**
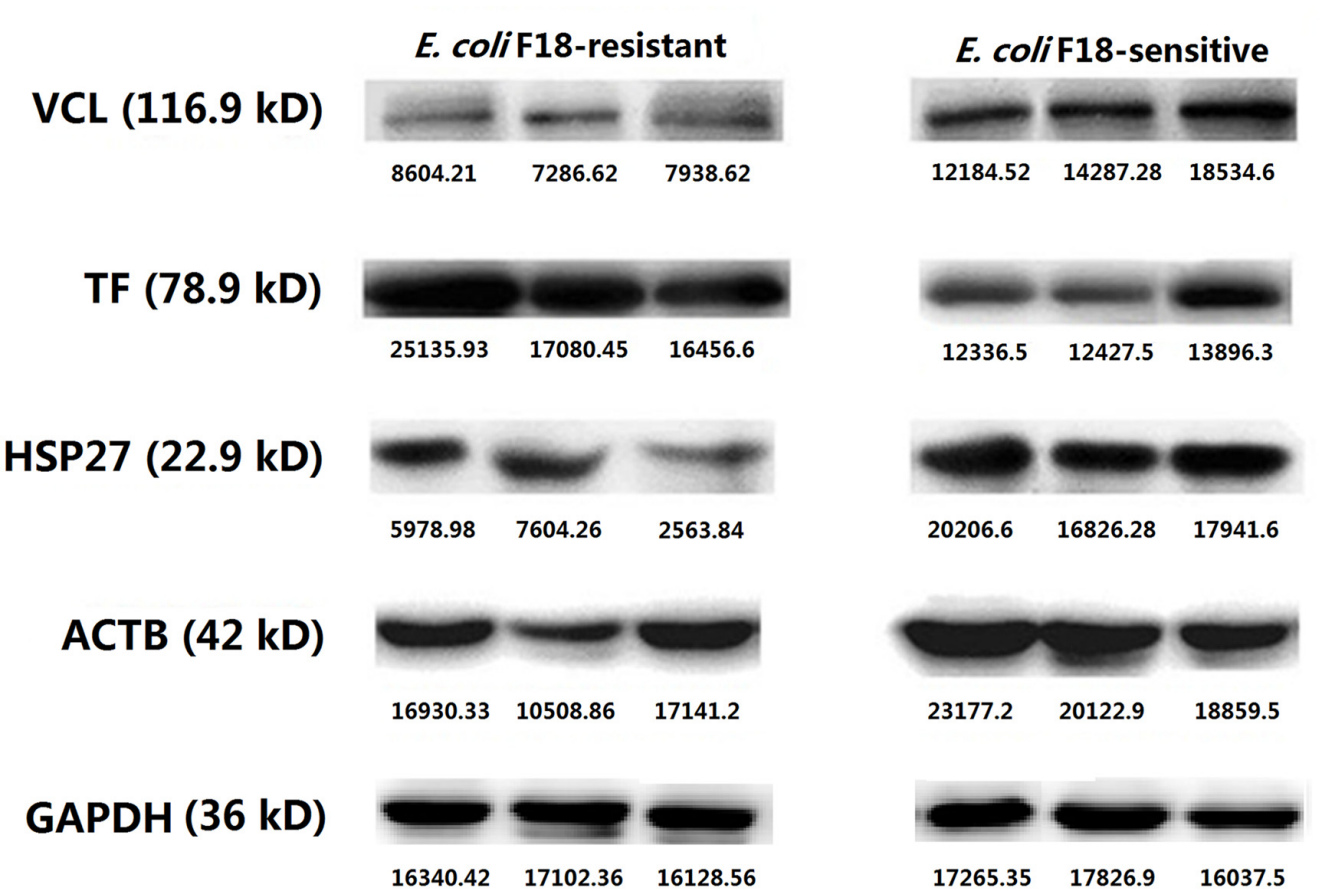
Differential expression of VCL, TF, HSP27 and ACTB proteins in duodenal tissue between *E*. *coli* F18-resistant and -susceptible groups, analyzed by western blot. Each image shows three samples from individual animals.

Based on the bioinformatics analysis of interactions between differentially expressed proteins, we biochemically screened several important proteins related to *E*. *coli* F18 infection, including upregulated transferrin (TF) from the *E*. *coli* F18-resistant group, and upregulated vinculin (VCL), actin beta (ACTB) and heat shock protein 27 kDa (HSP27) from the *E*. *coli* F18-susceptible group. In real-time PCR analysis (Fig 6), it was obvious that expression of TF mRNA in tissue from the *E*. *coli* F18-resistant group is significantly higher than in tissue from the susceptible group (*P* < 0.05), while the expression levels of VCL, ACTB and HSP27 were significantly higher in the *E*. *coli* F18- susceptible group (*P* < 0.05). Western blot analysis (Fig 7) was consistent with these qPCR results. These data confirm the accuracy of the 2-DE.

## Discussion

This study investigated the *E*. *coli* F18-susceptiblity status of complete sib-pair healthy individuals with extreme phenotypes from a Sutai pig resource colony, using an alvine epithelial cell adhesion assay system. Sib pairs were matched for factors including body weight, hair color, growth performance and genetic background as far as possible, and consistency of the feeding environment was maintained in order to minimize the number of differentially expressed proteins. Screening by 2-DE revealed only 20 differentially expressed proteins; sixteen were identified by mass spectral analysis. Excluding redundant proteins, only 12 significant proteins remained, including ACTB, VCL, HSP27 and TF, which were validated by Western blotting. On functional analysis, these 12 significant proteins were found to be involved mainly in cell adhesion and immune responses. Weaned piglet morbidity depends critically on *E*. *coli* F18-specific receptor expression in the intestinal epithelial cell mucosa. Mouricout et al. proposed that the biochemical structures of the *E*. *coli* F18 receptor comprised glycoprotein or glucosamines [16]. The results of this present study indicated that cell adhesion function-related proteins were represented among the differentially expressed proteins, with upregulated expression of ribosomal protein SA (RPSA) in the *E*. *coli* F18-resistant group and upregulated expression of ACTB and vinculin (VCL) in the susceptible group.

RPSA, also known as the 37 kDa laminin receptor precursor (37LRP), the 67 kDa laminin receptor (LR) and ribophorin P40 (P40), is a multifunctional protein encoded by the 37LRP full-length gene [17]. LR is a transmembrane protein containing a variety of monosaccharides and is involved in intercellular mutual recognition and intracellular and extracellular information transfer. This protein also functions as the host cell surface receptor for laminin, prion protein and many viruses. LR upregulation is closely related to the occurrence of several tissue cancers [18–20]. However, previous studies showed that bacteria and bacterial toxins predominantly combine with glycolipids, with few interactions between the microorganisms and glycoproteins. It can be speculated that the significance of glycolipids lies in the membrane surface location of the polysaccharides of glycolipids, while glycoprotein glycans are located more distally. The majority of adhesion molecules bind to oligosaccharides containing Galβ4Glc. Occasionally, adhesion molecules combine with Galβ4Glc at the end of lactose ceramide, although core oligosaccharides are usually covered by other monosaccharides (such as blood group antigens) [21]. Coddens et al. showed that F18-fimbriated *E*. *coli* selectively interact with glycosphingolipids having blood group ABH determinants on the type 1 core and blood group A type 4 heptaglycosylceramide [22]. We therefore concluded that LR was not associated with formation of the *E*. *coli* F18 specific receptor; upregulation of the laminin receptor in the *E*. *coli* F18-resistant pig group in the present work was possibly caused by the high complexity of proteins expressed within the host cells.

The cytoskeletal proteins ACTB and vinculin (VCL) were upregulated in the *E*. *coli* F18-susceptible group in our analyses. Studies have shown that *Shigella* with the type III secretion system secretes a series of effector molecules after contact with host cells and interacts with the cell membrane to activate signal transduction pathways associated with cytoskeleton formation and arrangement. This results in rearrangement of the cytoskeleton and further invasion of host cells [23]. Susceptibility to *E*. *coli* F18 infections in pigs has been shown to be dependent on the presence of the F18 receptor (F18R) on the porcine intestinal epithelial cells surface [24,25]. However, further research is required to establish whether tight junctions preventing entry of pathogenic bacteria into intestinal epithelial cells of lower tissues can resist *E*. *coli* F18 infection.

Differential protein analysis in this study also revealed upregulation of transferrin (TF) in the *E*. *coli* F18-resistant group, and of heat stress proteins (HSPs) in the susceptible group. These two protein types are mainly involved in immune responses. The complete sib pairs in this study were not infected with *E*. *coli* F18 and did not have diarrhea or ED symptoms. The immune system is essentially inactive under these conditions and therefore, our findings indicated that TF and HSPs might have an effect on innate immune responses to *E*. *coli* F18 infection. Heat stress proteins, also known as heat shock proteins, are a group of highly conserved, soluble, intracellular proteins, which are synthesized rapidly under conditions of stress. However, these proteins are also expressed under normal conditions and account for 5–10% of the total cell protein content [26]. David et al. detected expression of HSPs, predominantly HSP27, in heart, liver, lung and other tissues of newborn piglets [27]. Similarly, HSP27 was detected in duodenal tissues of healthy weaned piglets in the present study. HSPs play an important physiological role in cell growth, development and differentiation, gene transcription, and in protein synthesis, folding and decomposition. HSPs are also critically involved in anti-infective immunity and autoimmunity. Studies have shown that HSPs interact with pathogens invading host cells to form antigenic peptides which are taken up by macrophages and presented by MHC class I molecules via the endogenous pathway, thus stimulating a CD8^+^ T cell response [28,29]. Furthermore, as a Gram-negative bacterium, *E*. *coli* F18 releases endotoxin (LPS) after death, which strongly activates the MAPK signaling pathway that regulates inflammatory reactions through a multi-stage kinase cascade [30]. For example, LPS activates the p38 pathway, which stimulates intracellular protein kinases such as MAPKAPK2/3 (MAPK activated protein kinase 2/3) and PRAK (p38 regulated/activated protein kinase). These members of the serine/threonine kinase family are phosphorylated and activated, resulting in HSP27 activation, which mediates cytoskeleton reconstruction and participates in cellular stress responses [31].

Transferrin (TF) is a single-chain glycoprotein. Grange et al. hypothesized that the complex of transferrin with cellular transferrin receptor acts as a specific receptor for the *E*. *coli* K88ab pilus [32]. Furthermore, many studies have indicated that the transferrin gene is related to *E*. *coli* K88ab and K88ac resistance [33–35]. Apart from its role in regulation of cell proliferation and the immune system, transferrin is also involved in iron transport and metabolism and has the ability to chelate iron. Iron is an important growth factor for many bacteria [36]. Therefore, transferrin exerts antibacterial and disease-resistance functions as a result of competition with bacteria for iron resources. We speculate that transferrin plays additional roles in the resistance of the piglet intestinal tract to *E*. *coli* F18.

## Conclusions

Differentially expressed proteins related to adhesion and immunity were identified in screens of *E*. *coli* F18-resistant and -susceptible groups of piglets. 2-DE and Western blot analysis identified four promising candidate proteins, VCL, ACTB, TF and HSP27, which may be related to *E*. *coli* F18 infection. Based on the physiological functions of these proteins, we proposed that transferrin (TF) was the most plausible candidate for the *E*. *coli* F18 receptor. In further work, we will study the function of these proteins by reconstructing the TALEN/CRISPR-Cas9 vector and establishing knock-out cell lines in porcine intestinal epithelial cells. Meanwhile further validation and analysis was performed by using the adhesion test for intestinal epithelial cells. Such analysis is required to elucidate the molecular mechanism of porcine *E*. *coli* F18 resistance for disease-resistant breeding.

## Supporting Information

S1 Fig Two-dimensional electrophoresis images of duodenal proteins from *E*. *coli* F18-resistant and -susceptible individuals. (DOC)

S1 Table Primer sequence for differentially expressed genes. The selected genes were identified by real-time PCR. The housekeeping gene, *GAPDH* was used as the internal control. The data were analyzed by cycle threshold (C(t)) method.
(DOC)

S2 Table Information list of differential points of individual duodenal tissues with quantitative ratio >1.5 between *E*. *coli* F18-resistant and -susceptible groups. Note: “-” denotes down-regulation; values in Group ID column used values of corresponding points in original gel images.
(DOC)

S3 Table Analysis of significant function of differential protein-corresponding differential genes (41 items). (DOC)

S4 Table Differential protein-corresponding differential genes-involved in significant signal transduction pathways (six items). (DOC)

S5 Table Interactions of differential proteins with upstream and downstream proteins in interaction network of differential proteins. Note: Degree refers to the extent of the interaction between any protein and other proteins. Among these, Indegree represents the number of upstream proteins regulating a protein (arrow points to the protein), Outdegree represents the number of downstream proteins regulated by a protein (arrow points from this protein to the other proteins); Degree is equal to the sum of Indegree and Outdegree, and the line segment between two proteins represents their interaction.
(DOC)
