# Supplementary material for: Proteomic Analysis of Duodenal Tissue from Escherichia coli F18-Resistant and -Susceptible Weaned Piglets
Source: PLoS One. 2015 Jun 8;10(6):e0127164. doi: 10.1371/journal.pone.0127164 (PMC4459693; doi:10.1371/journal.pone.0127164)
Supplement: S1 Fig — (DOC) [file pone.0127164.s001.doc]

**
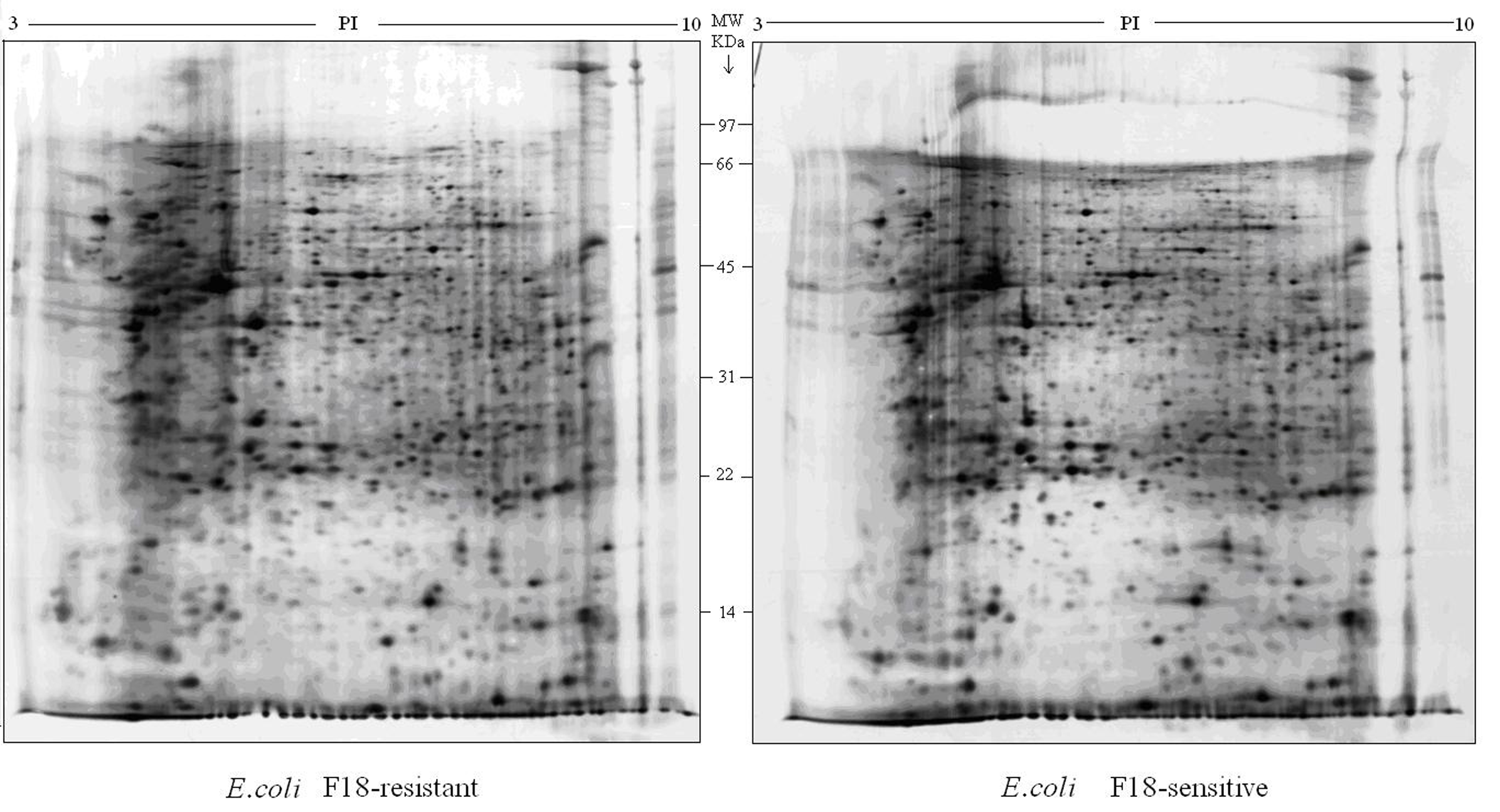
**

**S1 Fig.** Two-dimensional electrophoresis images of duodenal proteins from *E. coli* F18-resistant and -susceptible individuals.
