# Supplementary material for: Proteomic Analysis of Duodenal Tissue from Escherichia coli F18-Resistant and -Susceptible Weaned Piglets
Source: PLoS One. 2015 Jun 8;10(6):e0127164. doi: 10.1371/journal.pone.0127164 (PMC4459693; doi:10.1371/journal.pone.0127164)
Supplement: S1 Table — The selected genes were identified by real-time PCR. The housekeeping gene, GAPDH was used as the internal control. The data were analyzed by cycle threshold (C(t)) method. (DOC) [file pone.0127164.s002.doc]

**S1 Table. Primer sequence for differentially expressed genes**

| Gene | Sequence | Length of sequence (bp) | Accession no. |
| --- | --- | --- | --- |
| *TF* | F: 5'-CCCTGATAGCTCAAAGAATGAA-3'  R: 5'-GCCAAGGACACGCTTTCTCTC-3' | 152 | X12386.1 |
| *VCL* | F: 5'-GAACTCTGAGGATCCCAAG-3'  R: 5'-GATCCGGTATCCTGAGTCT-3' | 157 | AF165172.1 |
| *ACTB* | F: 5'-TGGCGCCCAGCACGATGAAG-3'  R: 5'-GATGGAGGGGCCGGACTCGT-3' | 149 | AY550069.1 |
| *HSP27* | F: 5'-TCGGAGATCCAGCAGAC-3'  R: 5'-GAGTGAAACACCGGGAAAT-3' | 163 | AY574049.1 |
| *GAPDH* | F: 5'-ACATCATCCCTGCTTCTACTGG-3'  R: 5'-CTCGGACGCCTGCTTCAC-3' | 188 | [AF017079](http://www.ncbi.nlm.nih.gov/nuccore/AF017079.1) |

The selected genes were identified by real-time PCR. The housekeeping gene, *GAPDH* was used as the internal control. The data were analyzed by cycle threshold (C(t)) method.
