# Supplementary material for: Proteomic Analysis of Duodenal Tissue from Escherichia coli F18-Resistant and -Susceptible Weaned Piglets
Source: PLoS One. 2015 Jun 8;10(6):e0127164. doi: 10.1371/journal.pone.0127164 (PMC4459693; doi:10.1371/journal.pone.0127164)
Supplement: S2 Table — Note: “-” denotes down-regulation; values in Group ID column used values of corresponding points in original gel images. (DOC) [file pone.0127164.s003.doc]

**S2 Table**. Information list of differential points of individual duodenal tissues with quantitative ratio >1.5 between *E. coli* F18-resistant and -susceptible groups

| Group ID | *E. coli* F18-resistant | *E. coli* F18-susceptible |
| --- | --- | --- |
| 515 | -2.33394 | 2.33394 |
| 579 | 1.77615 | -1.77615 |
| 677 | -2.39461 | 2.39461 |
| 684 | 1.51038 | -1.51038 |
| 730 | 1.88186 | -1.88186 |
| 1221 | 1.52664 | -1.52664 |
| 1352 | -1.56995 | 1.56995 |
| 1741 | 2.06623 | -2.06623 |
| 1912 | -2.10949 | 2.10949 |
| 1967 | 1.60262 | -1.60262 |
| 2074 | -1.55782 | 1.55782 |
| 2131 | -1.59203 | 1.59203 |
| 2198 | -1.55894 | 1.55894 |
| 2273 | 1.96878 | -1.96878 |
| 2287 | -2.85652 | 2.85652 |
| 2373 | 2.37319 | -2.37319 |
| 2403 | -1.58416 | 1.58416 |
| 2484 | 1.64586 | -1.64586 |
| 2558 | 1.64406 | -1.64406 |
| 2589 | -1.58920 | 1.58920 |

Note: “-” denotes down-regulation; values in Group ID column used values of corresponding points in original gel images.
