# Supplementary material for: Proteomic Analysis of Duodenal Tissue from Escherichia coli F18-Resistant and -Susceptible Weaned Piglets
Source: PLoS One. 2015 Jun 8;10(6):e0127164. doi: 10.1371/journal.pone.0127164 (PMC4459693; doi:10.1371/journal.pone.0127164)
Supplement: S3 Table — (DOC) [file pone.0127164.s004.doc]

**S3 Table**. Analysis of significant function of differential protein-corresponding differential genes (41 items).

| GO_ID | GO_name | *P*-value | FDR | Enrichment |
| --- | --- | --- | --- | --- |
| GO:0006936 | Muscle contraction | 3.32E-07 | 2.85E-06 | 44.686508 |
| GO:0017158 | Regulation of calcium ion-dependent exocytosis | 0.0008908 | 0.0015302 | 1251.2222 |
| GO:0034394 | Protein localization at cell surface | 0.0008908 | 0.0015302 | 1251.2222 |
| GO:0042660 | Positive regulation of cell fate specification | 0.0008908 | 0.0015302 | 1251.2222 |
| GO:0051659 | Maintenance of mitochondrion location | 0.0008908 | 0.0015302 | 1251.2222 |
| GO:0009991 | Response to extracellular stimulus | 0.0017812 | 0.0016999 | 625.61111 |
| GO:0043297 | Apical junction assembly | 0.0017812 | 0.0016999 | 625.61111 |
| GO:0046668 | Regulation of retinal cell programmed cell death | 0.0017812 | 0.0016999 | 625.61111 |
| GO:0048545 | Response to steroid hormone stimulus | 0.0017812 | 0.0016999 | 625.61111 |
| GO:0006101 | Citrate metabolic process | 0.0026712 | 0.0020854 | 417.07407 |
| GO:0043069 | Negative regulation of programmed cell death | 0.0026712 | 0.0020854 | 417.07407 |
| GO:0021940 | Positive regulation of granule cell precursor proliferation | 0.0035609 | 0.0023522 | 312.80556 |
| GO:0055003 | Cardiac myofibril assembly | 0.0044502 | 0.0025476 | 250.24444 |
| GO:0060045 | Positive regulation of cardiac muscle cell proliferation | 0.0044502 | 0.0025476 | 250.24444 |
| GO:0048741 | Skeletal muscle fiber development | 0.0053391 | 0.0026971 | 208.53704 |
| GO:0060047 | Heart contraction | 0.0053391 | 0.0026971 | 208.53704 |
| GO:0009612 | Response to mechanical stimulus | 0.0062276 | 0.002815 | 178.74603 |
| GO:0043536 | Positive regulation of blood vessel endothelial cell migration | 0.0062276 | 0.002815 | 178.74603 |
| GO:0002009 | Morphogenesis of an epithelium | 0.0071158 | 0.0029105 | 156.40278 |
| GO:0009267 | Cellular response to starvation | 0.0071158 | 0.0029105 | 156.40278 |
| GO:0031032 | Actomyosin structure organization | 0.0071158 | 0.0029105 | 156.40278 |
| GO:0051726 | Regulation of cell cycle | 0.0080035 | 0.0031247 | 139.02469 |
| GO:0030032 | Lamellipodium assembly | 0.008891 | 0.0033202 | 125.12222 |
| GO:0043537 | Negative regulation of blood vessel endothelial cell migration | 0.009778 | 0.0034993 | 113.74747 |
| GO:0010001 | Glial cell differentiation | 0.0106647 | 0.003664 | 104.26852 |
| GO:0000186 | Activation of MAPKK activity | 0.011551 | 0.003816 | 96.247863 |
| GO:0030048 | Actin filament-based movement | 0.0142076 | 0.0044052 | 78.201389 |
| GO:0006700 | C21-steroid hormone biosynthetic process | 0.0177446 | 0.0050856 | 62.561111 |
| GO:0060048 | Cardiac muscle contraction | 0.0186279 | 0.0052398 | 59.582011 |
| GO:0001934 | Positive regulation of protein amino acid phosphorylation | 0.02392 | 0.0060557 | 46.341564 |
| GO:0051592 | Response to calcium ion | 0.02392 | 0.0060557 | 46.341564 |
| GO:0045471 | Response to ethanol | 0.0274406 | 0.0065138 | 40.362007 |
| GO:0006879 | Cellular iron ion homeostasis | 0.0283198 | 0.0066194 | 39.100694 |
| GO:0045766 | Positive regulation of angiogenesis | 0.0283198 | 0.0066194 | 39.100694 |
| GO:0050679 | Positive regulation of epithelial cell proliferation | 0.0283198 | 0.0066194 | 39.100694 |
| GO:0006099 | Tricarboxylic acid cycle | 0.0291987 | 0.0067218 | 37.915825 |
| GO:0006826 | Iron ion transport | 0.0318331 | 0.007011 | 34.756173 |
| GO:0008543 | Fibroblast growth factor receptor signaling pathway | 0.0335875 | 0.0072124 | 32.926901 |
| GO:0051258 | Protein polymerization | 0.0405903 | 0.0082565 | 27.200483 |
| GO:0016049 | Cell growth | 0.0466985 | 0.0090813 | 23.607966 |
| GO:0030182 | Neuron differentiation | 0.0475696 | 0.009193 | 23.170782 |
