# Supplementary material for: Proteomic Analysis of Duodenal Tissue from Escherichia coli F18-Resistant and -Susceptible Weaned Piglets
Source: PLoS One. 2015 Jun 8;10(6):e0127164. doi: 10.1371/journal.pone.0127164 (PMC4459693; doi:10.1371/journal.pone.0127164)
Supplement: S4 Table — (DOC) [file pone.0127164.s005.doc]

**S4 Table**. Differential protein-corresponding differential genes-involved in significant signal transduction pathways (six items)

| Path_ID | Path_name | *P*-value | FDR | Enrichment |
| --- | --- | --- | --- | --- |
| 4810 | Regulation of actin cytoskeleton | 0.0002316 | 0.0010621 | 31.183642 |
| 4520 | Adherens junction | 0.0010749 | 0.0024651 | 58.31746 |
| 4670 | Leukocyte transendothelial migration | 0.0025509 | 0.0038999 | 37.734827 |
| 4510 | Focal adhesion | 0.0072164 | 0.0082744 | 22.229923 |
| 630 | Glyoxylate and dicarboxylate metabolism | 0.0133224 | 0.0122206 | 149.68148 |
| 20 | Citrate cycle (TCA cycle) | 0.0283199 | 0.0204264 | 70.163194 |
