# Supplementary material for: Proteomic Analysis of Duodenal Tissue from Escherichia coli F18-Resistant and -Susceptible Weaned Piglets
Source: PLoS One. 2015 Jun 8;10(6):e0127164. doi: 10.1371/journal.pone.0127164 (PMC4459693; doi:10.1371/journal.pone.0127164)
Supplement: S5 Table — Note: Degree refers to the extent of the interaction between any protein and other proteins. Among these, Indegree represents the number of upstream proteins regulating a protein (arrow points to the protein), Outdegree represents the number of downstream proteins regulated by a protein (arrow points from this protein to the other proteins); Degree is equal to the sum of Indegree and Outdegree, and the line segment between two proteins represents their interaction. (DOC) [file pone.0127164.s006.doc]

**S5 Table**. Interactions of differential proteins with upstream and downstream proteins in interaction network of differential proteins

| Protein | Description | Degree | Indegree | Outdegree |
| --- | --- | --- | --- | --- |
| VCL | Vinculin | 4 | 2 | 2 |
| ACO2 | Aconitase 2, mitochondrial | 4 | 2 | 2 |
| ACTC1 | Actin, alpha, cardiac muscle 1 | 2 | 1 | 1 |
| Hsp27 | Heat shock 27kDa protein 1 | 2 | 2 | 0 |
| LOC100151886 | Similar to collapsin response mediator protein-2A | 3 | 3 | 0 |

Note: Degree refers to the extent of the interaction between any protein and other proteins. Among these, Indegree represents the number of upstream proteins regulating a protein (arrow points to the protein), Outdegree represents the number of downstream proteins regulated by a protein (arrow points from this protein to the other proteins); Degree is equal to the sum of Indegree and Outdegree, and the line segment between two proteins represents their interaction.
